# Automated Features, Algorithms, and Technologies of Electronic Early Warning/Track-and-Trigger Systems: Systematic Review

**DOI:** 10.2196/58233

**Published:** 2026-08-10

**Authors:** Sharareh Rostam Niakan Kalhori, Mostafa Haghi, Masresha Derese Tegegne, Viktor MG Sobotta, Paulo Haas, Nagarajan Ganapathy, Thomas M Deserno

**Affiliations:** 1 Department of Health Information Management and Medical Informatics, School of Allied Medical Sciences, Tehran University of Medical Sciences, No.17, Bastani Parizi Alley, Qods Street, Tehran, 1417744361, Iran, 98 2188983025; 2 Peter L. Reichertz Institute for Medical Informatics of TU Braunschweig and Hannover Medical School, Braunschweig, Germany; 3 ECLECTX Team, Institute for Computer Engineering, Heidelberg University, Heidelberg, Germany; 4 Department of Biomedical Engineering, Indian Institute of Technology Hyderabad, Sangareddy, Telangana, 502284, India

**Keywords:** early warning, clinical deterioration, artificial intelligence, automation, patient monitoring, vital signs, PRISMA

## Abstract

**Background:**

Electronic early warning/track-and-trigger systems (EW/TTS) are crucial for patient monitoring, detecting clinical deterioration (CD), and activating rapid response teams. Understanding the current level of automation in EW/TTS is essential.

**Objective:**

This study aimed to provide a comprehensive overview and critical assessment of electronic EW/TTS, including automated features, algorithms, and technologies, following a published registered study protocol.

**Methods:**

Based on the PRISMA (Preferred Reporting Items for Systematic Reviews and Meta-Analyses) guidelines, we included studies from PubMed, Web of Science, and Scopus published between January 2010 and December 2025 describing EW/TTS applied in real-world settings, and electronic systems for CD detection. We excluded studies outside the clinical context or those that used manual scoring charts. We applied a descriptive narrative approach and a methodological quality assessment according to the Joanna Briggs Institute Critical Appraisal Checklist.

**Results:**

After removing outliers and duplicates, the query returned 1181 studies. The selected studies (n=43) reported CD as the primary objective in 24 of 44 (54.5%) reported primary objectives, with ICU transfer in 16 of 68 (23.5%) reported secondary objectives, and mortality prediction in 10 of 68 (14.7%) reported secondary objectives. EW/TTS primarily relied on vital signs and assessment scores, accounting for 42 of 67 (62.7%) reported clinical indexes to detect and predict CD effectively. Among the included systems, 18 of 43 (41.9%) had a measured automation level, 11 of 43 (25.6%) had a managed automation level, and 7 of 43 (16.3%) had a defined automation level. The studies focused on several technological domains, with a strong emphasis on data analytics (24/43, 55.8%) and hardware technologies (7/43, 16.3%). Predictive algorithms, including statistical and machine learning approaches, were used in 11 of 43 (25.6%) systems. Interoperable connectivity was reported in 30 of 43 (69.8%) systems, including connectivity with electronic health records, wearable devices, and communication platforms such as Ascom Unite, as well as integrations using standards such as Health Level Seven Fast Healthcare Interoperability Resource and Health Level Seven. Evaluations of the systems showed earlier warning (14/70, 20%), higher accuracy (12/70, 17.1%), and lower specificity (9/70, 12.9%) as the main reported outcomes. Electronic EW/TTS were most prevalent in the United States (15/43, 34.9%), the United Kingdom (6/43, 14%), and the Netherlands (6/43, 14%).

**Conclusions:**

Current EW/TTS systems implemented a measured level of automation and primarily focused on patient monitoring in hospital surgery wards. More than half of EW/TTS featured data exchange capabilities and connectivity with other systems. Reported outcomes of EW/TTS included early warning, high accuracy, and lower specificity. However, the included evidence was limited by heterogeneous prediction targets, inconsistent performance metrics and time horizons, and poor reporting of development history and system failure. Using clinically validated wearable devices and establishing a standardized data collection framework may further improve system accuracy and reliability.

## Introduction

Early Warning/Track-and-Trigger Systems (EW/TTS) identify patients at risk of physiological deterioration and alert appropriate medical teams in hospital wards or intensive care units (ICUs) [1,2]. These systems are part of hospitals’ efforts to identify patients with clinical deterioration (CD) early [3]. These patients are prone to worsening, leading to an increased risk of adverse events (AEs) such as organ dysfunction, prolonged hospital stay, disability, or death [4]. Vital signs (VS) provide essential information for assessing patients’ clinical status, as 85% of severe AE cases result from abnormal VS [5,6]. Thus, any acute deviation from a patient’s normal range of VS increases the risk of CD and AE [4]. Physicians and care providers consider the physiological shift from one clinical state to a worse status as CD. To record a quick and simple assessment of an individual’s level of consciousness, especially in an emergency, physicians apply the alert, verbal, pain, and unresponsive (AVPU) method.

In 1997, Morgan et al [7] introduced a manual Early Warning Score (EWS) chart to detect CD. This chart calculates EWS using five critical VS: systolic blood pressure, heart rate (HR), respiratory rate (RR), body temperature, and blood oxygen saturation (SpO_2_) [8]. To use the chart and calculate EWS, nurses scale and aggregate VS based on patients’ records [9]. Later, the Royal College of Physicians, in collaboration with National Health Services in the United Kingdom, gradually substituted the manual chart with electronic versions [10] due to limitations such as deteriorated patient misidentification, mis-scoring, particularly in acute cases, delay in data recording, nurses’ difficulties in score trends analysis and interpretation [11], delays in CD detection, interruptions, stimuli from multiple alarms, and delays in rapid response team (RRT) activation [12]. Patient safety is not compromised [13]; hence, the electronic EW/TTS evolved gradually to improve existing systems and overcome the limitations. Now, EW/TTS automatically monitors patients’ VS and alerts health professionals at the moment of CD. EW/TTS consists of afferent and efferent limbs and mainly receives input from bedside VS observations. The afferent limb generates the EWS for CD detection, and the efferent limb represents the appropriate response and actions of the RRT [11].

Electronic EW/TTS offers advantages in practice. They provide data entry with bedside capture of VS using a handheld device with automatic calculation of EW/TTS and embedded alerts, with a higher level of accuracy from 81% to 100%, and significantly improve patient-centered clinical outcomes [14,15]. Furthermore, automated alerts increase timely clinical attendance at patients’ bedside for EWS > 5 from 67% to 96% [15]. They are widely used in the United States, United Kingdom, and Australia [16] and provide faster and more accurate calculations of EW/TTS compared to a manual method [11]. Despite these advantages, several limitations of current electronic EW/TTS have prompted further improvement. Patients prefer not to be continuously attached to an unwieldy VS monitoring machine. Only 16% of patients in hospital wards remain connected to their monitors for a 72-hour study period, while the rest desire greater mobility and comfort [17,18]. Developing systems that use new technologies, such as ambient sensors, may mitigate this problem [19]. The limitations of wearable devices and the emergence of new technologies inspire the creation of a private diagnostic space using an unobtrusive sensor approach for VS monitoring and CD detection [20]. Furthermore, current versions of electronic EW/TTS require the continuous presence of clinicians, and thus, the systems still need a higher level of automation [21]. Only a few devices are capable of measuring all VS automatically. However, none have yet been tested adequately or mature enough for widespread acceptance [11].

Besides VS, other physiological data also contribute to the detection of CD [22-24]. A system requirement considers clinical factors to improve the afferent limb. The clinical factors and data from ventilators, dialysis machines, other devices, and VS feed the system [12]. Concurrently, the efferent limb of electronic EW/TTS needs enhancement for RRT’s timely activation. Wood, Chaboyer, and Carr revealed the inconsistent functionality of current electronic EW/TTS for both limbs [13] because they focus on a single set of observations at one point in time, rather than observational trends, and compare datasets over a period [25]. Therefore, current systems may not ensure patient safety due to misidentification of CD events.

Modern technology and system automation have further advanced EW/TTS to overcome current limitations. Although automation in health monitoring can provide numerous benefits, there are challenges and considerations, including data privacy, security, ethical concerns, patient safety, and human oversight in critical decision-making [26]. Consequently, the level of automation needs harmonization with other essential prerequisites.

It is important to use a structured framework that distinguishes early, partially implemented automation from more advanced, measurable, and continuously improving systems to understand the maturity of automation in EW/TTS. This review used the capability maturity model (CMM), as modified by Informatics-Savvy Health Organizations (ISHO), which delineates 6 tiers of digital health maturity: absent, initial, managed, defined, measured, and optimized [27].

In a previous requirement analysis, we identified that an intelligent EW/TTS requires five subsystems to effectively monitor patients, detect cardiac arrests, and alert the RRT: (1) direct patient monitoring, (2) electronic health record (EHR), (3) clinical decision support, (4) remote patient monitoring, and (5) dashboards and registries [21]. Each subsystem needs appropriate technologies for adequate patient monitoring, cardiac arrest detection, and automatic alarming of the RRT. To this end, we need to know the current level of available EW/TTS from a technical perspective. So far, reviews have focused on manual systems of score calculation for specific patient groups, particularly in hospital settings [16]. These reviews mainly target the efficiency, strengths, and limitations of manual EWS for enhancing care levels and preventing AE [13]. The introductory review by Despins on automated CD detection in the ICU does not include electronic EW/TTS to detect CD and activate RRT [12].

This study aims to conduct a comprehensive review of the in-depth application of EW/TTS for different target groups of patients in both private spaces and hospital settings. In previous work, we proposed a registered study protocol to systematically assess the current status of EW/TTS in terms of automation capabilities, applied algorithms, and performance [28]. Furthermore, we address the functionality of current systems and justify the suitability of available EW/TTS in terms of automation and intelligence.

## Methods

### Study Design

We published the details of the study and the design rationale in a registered study protocol [28] and in the PROSPERO database (registration number: CRD322838). We conducted the review according to the PRISMA (Preferred Reporting Items for Systematic Reviews and Meta-Analyses) guidelines and flowchart (Checklist 1) [29] to answer the following questions:

1. Which automated features does the EW/TTS consider?
2. Which predictive algorithms, including statistical and machine learning approaches, are applied?
3. Which technologies and communication standards do EW/TTS use?
4. Which settings host EW/TTS (hospital wards or others)?
5. Which clinical parameters or VS do EW/TTS monitor?
6. What are the primary and secondary purposes of EW/TTS?
7. Does EW/TTS analyze data for CD detection or prediction?
8. What are the results of evaluating EW/TTS and its algorithms?

### Search Strategy and Selection Criteria

We searched the databases PubMed, Web of Science, and Scopus without restrictions on the document type. We used the MeSH to determine the relevant keywords and their synonyms. Two clinical specialists in the areas of emergency medicine and medical informatics approved the search terms. We composed English terms to search the databases by title, abstract, and keywords to find relevant studies published between January 2010 and December 2025. The search strategy covered two main concept areas relevant to the technical details of EW/TTS: technological and clinical terminology. We provided further details in the registered study protocol (Multimedia Appendix 1) [28].

### Eligibility Criteria

We predefined the eligibility criteria presented in Textbox 1 in the registered study protocol [28] for the focused analysis of automated features, algorithms, and technologies of EW/TTS.

Textbox 1. **Inclusion and exclusion criteria.**

**Inclusion criteria**

- All original studies are in the English language.
- The studies that report electronic systems for CD detection via human vital sign monitoring.
- The studies that report electronic EW/TTS have already been developed and applied in the real world.
- The reported EW/TTSs are applied either for physiological data monitoring or other risk factors of CD detection or monitoring.
- The reported EW/TTS in any setting, including hospitals, clinics, or smart ambients.
- The reported EW/TTS across all types, including bed-connected systems, wearable devices, and unobtrusive sensing technologies.

**Exclusion criteria**

- Studies conducted outside the medical or health care context.
- The reports of EW scoring charts that manually calculate risk scores such as MEWS, EWS, PEWS, and MEWT.
- Studies published in languages other than English.
- Studies available only as abstracts.
- Study protocols and ongoing work descriptions were excluded unless they described an existing EW/TTS and provided sufficient technical detail relevant to this review.
- Studies that do not provide any details on the system’s technologies, algorithms, or features.
- Studies that report only models, system designs, or prototypes.

### Selection Process

After searching the databases, we transferred the results into an EndNote library and removed duplicates. We imported the remaining records into Covidence [30] to support systematic screening. Four authors (SRNK, MDT, PH, and VMGS) with expertise in Medical Informatics and Biomedical Engineering independently reviewed the titles and abstracts to identify relevant studies. Subsequently, they assessed the full texts against the predefined inclusion and exclusion criteria following the PRISMA framework. We summarized the selection process in the PRISMA flow diagram (Figure S1 in Multimedia Appendix 2). During data extraction, the reviewers developed an Excel-based data extraction sheet that included variables reflecting the study aims and key features and functions of EW/TTSs. Two additional authors (MH and NG) contributed to data extraction and helped resolve inconsistencies in the extracted data through discussion until consensus was reached.

### Data Extraction and Analysis

After completing the selection process, four authors (SRNK, MDT, PH, and VMGS) extracted the relevant parameters from the included studies. These parameters included the first author’s last name, title, publication year, country, study duration, study design, study population, hospital ward, primary and secondary objectives, clinical index, automation level, technology type, predictive algorithms, including statistical and machine learning approaches, and reported outcomes.

The extracted data were analyzed using a descriptive narrative approach, with results organized according to the review objectives. To assess automation levels, we adopted the CMM, as adapted by ISHO [27]. This framework evaluates the stages of maturity of digital health systems and defines six levels: absent, initial, managed, defined, measured, and optimized. Their operational definitions and decision rules are summarized in Table 1. The six levels are defined as follows: level 1, absent (no meaningful automation in data acquisition, analysis, or alerting); level 2, initial (limited, isolated automation without an integrated workflow); level 3, managed (automation implemented in practice without standardization or performance tracking); level 4, defined (structured and integrated automation workflow with limited measurement or feedback); level 5, measured (automation supported by explicit evaluation or performance monitoring); and level 6, optimized (automation continuously refined through feedback or quality improvement). The adapted CMM was used to assess workflow and system automation maturity rather than the sophistication of the underlying analytic method. To improve transparency and reproducibility, we operationalized the adapted CMM maturity levels using explicit decision rules based on the degree of automation reported for each EW/TTS. Classification considered whether the system included automated data acquisition, automated data analysis and/or score calculation, automated alert generation or escalation support, integration with other digital systems, and evidence of performance monitoring or continuous improvement. Reviewers independently assigned each EW/TTS to one maturity level using these criteria and the operational definitions presented in Table 1. Disagreements were resolved through discussion until consensus was reached. When a study did not provide sufficient evidence to support a higher maturity level, the lower level was assigned based on the available evidence. Furthermore, we grouped the applied technologies into six mutually exclusive domains: Data Analytics, Hardware, Data Collection, Communication, Software, and Unimplemented Systems; their operational definitions and representative examples are summarized in Table 2.

**Table 1.** Operational definitions and decision rules for adapted capability maturity model maturity levels used in early warning/track-and-trigger systems classification.

Maturity level	Operational definition or decision rule	Typical EW/TTS^a^ characteristics	Representative examples
Absent	No meaningful automation in data acquisition, analysis, or alerting.	Manual or minimally digitized workflow.	Pediatric rapid response system [31]: manual human-triggered activation with no electronic monitoring, scoring, or alerting.
Initial	Limited, isolated automation without an integrated workflow.	Standalone digital function; fragmented automation.	Wearable heart attack detection system [32]: a standalone ECG^b^ chest belt wearable system operating independently from clinical workflows.
Managed	Automation is implemented in practice without standardization or performance tracking.	Operational system; partial workflow integration.	ViSi Mobile and HealthPatch [33]: continuous wearable vital sign monitoring implemented on general wards as a pilot project, but has not yet been integrated into the standardized clinical workflow.
Defined	Structured and integrated automation workflow with limited measurement or feedback.	Organized workflow; some interoperability or standardization.	Two-tiered clinical warning system [3]: structured architecture featuring EMR^c^-based risk identification with wireless sensor monitoring and automated nurse notification, but the real-time event detection remains at the feasibility stage.
Measured	Automation supported by explicit evaluation or performance monitoring.	Integrated system with tracked outcomes or metrics.	Advance alert monitor [34]: automated EHR^d^-based predictive model implemented across 19 hospitals with remote monitoring, featuring explicit outcome evaluation, including reduced mortality and ICU^e^ admissions.
Optimized	Automation is continuously refined through feedback or quality improvement.	Mature system with iterative optimization.	ESCALATION system [35]: evidence-based pediatric early warning system developed using an iterative methodology, stakeholder coproduction, and prototyping, incorporating refinement through feedback from diverse stakeholder groups.

a EW/TTS: early warning/track-and-trigger systems.

b ECG: electrocardiogram.

c EMR: electronic medical record.

d EHR: electronic health record.

e ICU: intensive care unit.

**Table 2.** Technology domains used in early warning/track-and-trigger systems classification.

Technology domain	Operational definition and decision rule	Typical EW/TTS^a^ characteristics	Representative examples
Data analytics	Technologies used for data processing, score calculation, prediction, visualization, or decision support.	Risk scoring, prediction, dashboards, trend analysis.	Advance alert monitor [34]: automated EHR^b^-based risk model
Hardware	Physical monitoring or computing devices used in EW/TTS.	Wearables, patches, bedside monitors, sensors.	ViSi Mobile & HealthPatch [33]: wearable monitoring hardware
Data collection	Technologies used to capture or acquire patient data before analysis.	ECG^c^ capture, sensor input, vital-sign acquisition.	None among included studies.
Communication	Technologies supporting alerts, messaging, connectivity, or information transfer.	Alert transmission, escalation, interoperability, wireless exchange.	SEND [36,37]: digital notification and alerting system
Software	Digital applications or platform functions supporting EW/TTS workflow.	Mobile apps, dashboards, APIs, digital charting.	Abuelómetro [38]: mobile application
Unimplemented systems	Proposed, conceptual, or feasibility-stage systems not yet deployed in routine care.	Prototype, pilot, simulation, feasibility-stage workflow.	Two-tiered clinical warning system [3]: feasibility-stage architecture

a EW/TTS: early warning/track-and-trigger systems.

b EHR: electronic health record.

c ECG: electrocardiogram.

### Quality Assessment

We assessed the methodological quality of the included studies using the Joanna Briggs Institute (JBI) critical appraisal checklist with 8 main items [39]. We adapted the JBI case report checklist, which considers the step-by-step cycle of system development (Multimedia Appendix 3).

## Results

### Overview

**Figure 1.**
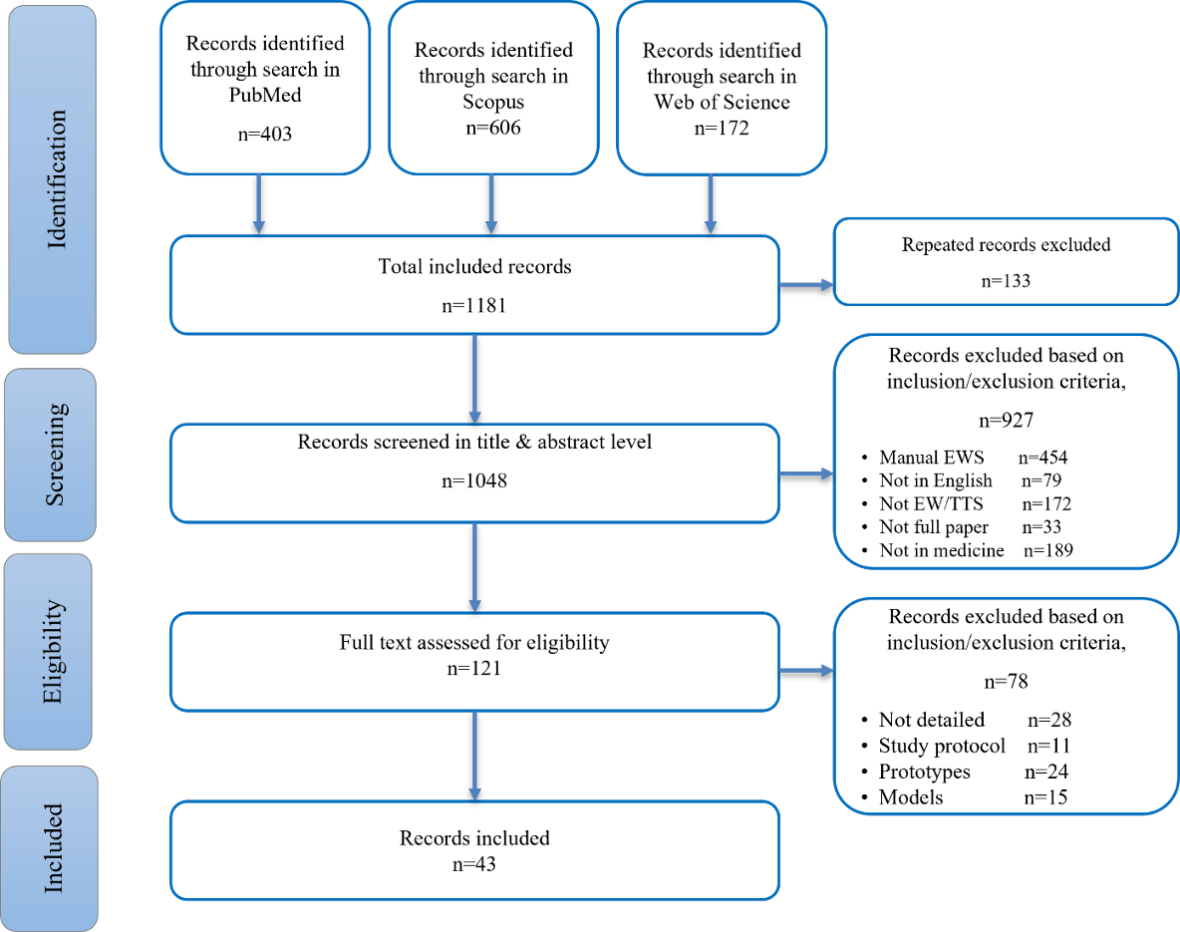
PRISMA (Preferred Reporting Items for Systematic Reviews and Meta-Analyses) flow diagram. EW/TTS: early warning/track-and-trigger systems.

Using PRISMA [29], we identified 1181 studies and removed 133 records due to duplication (Figure 1). We further excluded 927 records at the title and abstract level. We screened 121 full-text records, of which 43 studies [1,3,4,17,31-38,40-70] met the eligibility criteria (Figure S1 in Multimedia Appendix 2). We classified the studies according to the Study Design Classification (SDC) framework [71]. The analysis included descriptive studies (cross-sectional: 10/43, 23.3% [1,32,35,38,46,55,60-62,66], qualitative: 5/43, 11.6% [4,33,49,50,59]) and analytical studies. The latter consisted of experimental (randomized clinical trials: 3/43, 7% [36,37,56], nonrandomized clinical trials: 9/43, 20.9% [3,31,34,40,41,51,57,65,70]) and observational designs (cohort: 6/43 [17,42,45,48,53,67], 14%, retrospective: 10/43, 23.3% [43,44,47,52,54,58,63,64,68,69]). There were reports from 16 countries; 26 of 43 (60.5%) [1,33,34,36,37,40,43,45,46,48-50,53,55,57-63,68,69] studies were from the United States, United Kingdom, and the Netherlands. The studies ranged from 0.8 to 78 months, with an average of 22.8 months (Table 3).

**Table 3.** General characteristics of the studies.

Authors	Year	Country	Study design^a^	Study duration (month)	System name^b^	Chart^c^	Patient population^d^	Primary objective^e^	Secondary objective^f^	Connectivity^g^	Data analysis algorithm usage^h^
Youssef Ali Amer et al [40]	2020	Belgium, Netherlands	Cohort	24	NS	A	Cardiology, postsurgical dialysis	Score calculation	EWS error reduction	NS	HybridKNN-LS-SVM
Bassin et al [41]	2023	Australia	Non-RCT	28	BTF + DI	A	NS	CD	RRT activation, ICU transfer, Death, Length of stay	EHR	Logistic regression
Bell et al [42]	2021	Australia	Non-RCT	32	Deterioration Index (DI)	A	NS	CD, Adverse event	ICU transfer, Adverse events, Death	EHR	Logistic regression
Breteler et al [1]	2020	Netherlands	Cross-sectional	7	NS	A	Atrial fibrillation	Adverse event	Heart rate	None	None
Chowdhury et al [32]	2019	Qatar	Cross-sectional	24	NS	A	NS	Adverse event	Heart rate	None	SVM (Polynomial Kernel)
Durán-Vega et al [38]	2019	Mexico	Cross-sectional	NS	Abuelómetro	A	NS	Nurse satisfaction	Clinical intervention	Wearable device	None
Edelson et al [43]	2024	United States	Retrospective	48	eCART	A	NS	CD	ICU transfer, Death, EWS error reduction	EHR	eCART
Ehara et al [44]	2024	Japan	Retrospective	3	VSI	A	NS	CD	ICU transfer	EHR	Multivariate probabilistic model
Emmanuel and Torres [45]	2018	United States	Retrospective	5	EPIC	C	NS	Clinical intervention	Score calculation	None	None
Escobar et al [34]	2020	United States	Non-RCT	42	AAM Advance Alert Monitor	A	NS	CD	ICU transfer, Length of stay	EHR	Logistic regression
Fletcher et al [46]	2017	United States	Cross-sectional	5	RRT	A	NS	ICU transfer	Death	NS	None
Gill et al [35]	2022	Australia	Cross-sectional	4	Escalation	C	NS	CD	Clinical intervention	None	None
González et al [47]	2025	Mexico	Retrospective	40	NEURO-EWS	A	Neurological conditions	CD	Death, ICU transfer, Clinical intervention	EHR	Logistic regression
Gorham et al [48]	2020	United States	Retrospective	78	VRI	C	Hospitalized	CD	Score calculation	None	None
Hackmann et al [3]	2011	United States	Retrospective	38	NS	A	NS	ICU transfer	Clinical intervention	EHR	None
Hanson et al [31]	2010	United States	Non-RCT	45	PRRS	C	NS	CD	Death	NS	None
Hobensack et al [49]	2024	United States	Qualitative	7	Concern	A	NS	CD	Early sepsis	EHR with FHIR	None, Only rule-based score calculation
Howard et al [50]	2022	United States	Qualitative	22	Decision System	A	NS	Death	Length of stay	EHR^i^	None
Huete-Garcia & Rodriguez-Lopez [51]	2024	Spain	Retrospective	73	AI-mNEWS2-Lab	A	NS	CD	ICU transfer, Adverse events, Death, Length of stay, EWS error reduction	EHR	Logistic regression, Decision trees, Neural networks, Ensemble models
Huff et al [4]	2019	United States	Qualitative	4	VAS	A	Chronic conditions	Early sepsis	CD	None	None
Itelman et al 52]	2022	Israel	Retrospective	NS	NS	A	Hospitalized	CD	NS	None	None
Jerry et al [53]	2025	Netherlands	Cohort	8	ViQtor	A	NS	CD	Adverse events, ICU transfer, RRT activation, Clinical intervention	EHR	None
Lakshman et al [54]	2025	India	Retrospective	6	S-MEWS	A	NS	CD	ICU transfer	Wearable device	None
Lang et al [55]	2019	United Kingdom	Cross-sectional	9	eObs	A	Hospitalized	Nurse satisfaction	NS	None	None
Lee et al [56]	2017	ROK	RCT	3	MES	A	Pulmonary disease	Clinical intervention	RRT activation	EHR	None
Padilla et al [57]	2025	United States	Non-RCT	18	E-MEWS	PW	NS	CD	Death, EWS error reduction, Adverse events	EHR	None
Picker et al [58]	2017	United States	Retrospective	12	NS	A	Palliative care	Death	Clinical intervention	EHR	None
Posthuma et al [59]	2020	United Kingdom, France, Netherlands	Qualitative	NS	Sensium Vitals	A	NS	CD	Clinical intervention	NS	None
Rossetti et al [60]	2021	United States	Cross-sectional	12	CONCERN CDS	A	NS	ICU transfer	Length of stay	EHR with FHIR	NLP, decision trees, and logistic regression
Schmidt et al [61]	2015	United Kingdom	Cross-sectional	84	EPSS VitalPAC	A	NS	Death	CD	EHR	None
Shields et al [62]	2016	United States	Cross-sectional	37	NS	PW	NS	Death	ICU transfer	EHR	None
Teheux et al [63]	2019	Netherlands	Cohort	47	Press	C	Cardio- pulmonary disease	ICU transfer	CD	EHR	None
Tomasi et al [64]	2020	Canada	Non-RCT	3	Bedside PEWS	C	NS	Nurse satisfaction	Score calculation	EHR	None
Tran et al [65]	2025	Canada	Cohort	12	VSM + EWS	A	NS	CD	ICU transfer, RRT activation, Nurse satisfaction	EHR with HL7	None, Only rule-based score calculation
Un et al [66]	2021	Hong Kong	Cross-sectional	0.8	NS	A	SARS- COVID-19	CD	Score calculation	NS	None
Verma et al [67]	2024	Canada	Non-RCT	67	CHARTwatch	A	NS	CD	Death, ICU transfer, Length of stay	EHR	Time-aware MARS model
Weenk et al [33]	2017	Netherlands	Qualitative	4	ViSi Mobile & Health Patch	A	Internal medicine, Surgical ward	CD	Score calculation	Wearable device	None
Weller et al [68]	2018	United States	Non-RCT	5	VSAS	A	Neurosurgical patients	RRT activation	ICU transfer	EHR with Ascom platform (Morrisville)	None
Wilson et al [69]	2016	United Kingdom	Cohort	12	eT&T	A	NS	CD	Score calculation	EHR	None
Wong et al [36]	2015	United Kingdom	RCT	9	SEND	A	NS	Score calculation	Heart rate	EHR with FHIR	None
Wong et al [37]	2024	United Kingdom	RCT	20	SEND	A	NS	CD	ICU transfer, Clinical intervention, Length of stay, Death	EHR	None, Only rule-based score calculation
Wu et al [17]	2021	Taiwan	Cohort	15	E-NEWS	A	Cancer, Cardio-vascular, Neurological disorders	CD	ICU transfer	EHR	None
Yadav et al [70]	2024	India	Non-RCT	3	Dozee-EWS	A	NS	CD	ICU transfer, EWS error reduction	Proprietary cloud platform (NS)	None, Only rule-based score calculation

a Study design (the type of study used to report or evaluate the EW/TTS): RCT: randomized clinical trial, non-RCT: nonrandomized clinical trial.

b System name (the name of the EW/TTS): NS: not specified.

c Chart (the type of chart or tool used to monitor and assess patients in three main groups): A: adult, C: children, PW: pregnant women.

d Patient population (the specific disease, condition, or patient group addressed by the EW/TTS): NS: not specified.

e Primary objective (The main goal or objective of the EW/TTS): NS: not specified, RRT = rapid response team, PEWS: pediatric early warning system.

f Secondary objective (Additional goals or objectives of the EW/TTS, if any): NS = not specified.

g Connectivity (If the EW/TTS is connected with other systems and the applied standard is reported, it is reported in a format such as EHR with HL7): EHR = electronic health record, HL7 = Health Level Seven, NS = not specified, None = system was not connected.

h Data analysis algorithm (predictive algorithms used in the EW/TTS, including statistical and machine learning approaches): Rule-based score calculation = automated score calculation based on predefined rules or thresholds, such as EWS tables.

i FHIR: Fast Healthcare Interoperability Resource.

### Electronic EW/TTS Characteristics

Different EWS charts for adults, pediatric patients, and pregnant women reflect each group’s physiological norms and clinical risks, ensuring accurate assessment and timely intervention. This differentiation enhances the sensitivity and specificity of monitoring, improving patient outcomes through tailored clinical responses [2,8,16,17,62]. We identified different participant groups across the included studies: adults older than 18 years (35/43, 81.3% [1,3,4,17,32-38,40-44,46,47,49-56,58-61,65-70]), children, patients younger than 18 years (6/43, 14% [31,35,45,48,63,64]), and pregnant women (2/43, 4.7% [57,62]). Most EW/TTS were applied in health monitoring in hospitals (39 of 46, 84.8% reported settings [1,3,4,17,31,33-37,41-46,48,50-54,56,57,59-65,67,69,70]). Health care providers use them primarily in hospitals: surgery wards 12 of 46 (26.2%) reported settings [1,4,40-43,46,47,49,53,54,65], general wards 10 of 46 (21.7%) reported settings [17,33,34,51,52,56,60,61,67,70], pediatric wards 6 of 46 (13%) reported settings [31,35,45,48,63,64], emergency wards 3 of 46 (6.5%) reported settings [3,50,69], and other wards including ICU, respiratory, maternal, oncology, and short-stay 8 of 46 (17.4%) [36,44,49,56,57,62] reported settings. In addition, 7 of 46 (15.2%) reported settings [32,38,40,46,58,66,68] applied in other settings (Figure S2 in Multimedia Appendix 2); one study applied VS monitoring for CD detection in cars [32] while another applied it in older adult care residences and hospices [38].

**Figure 2.**
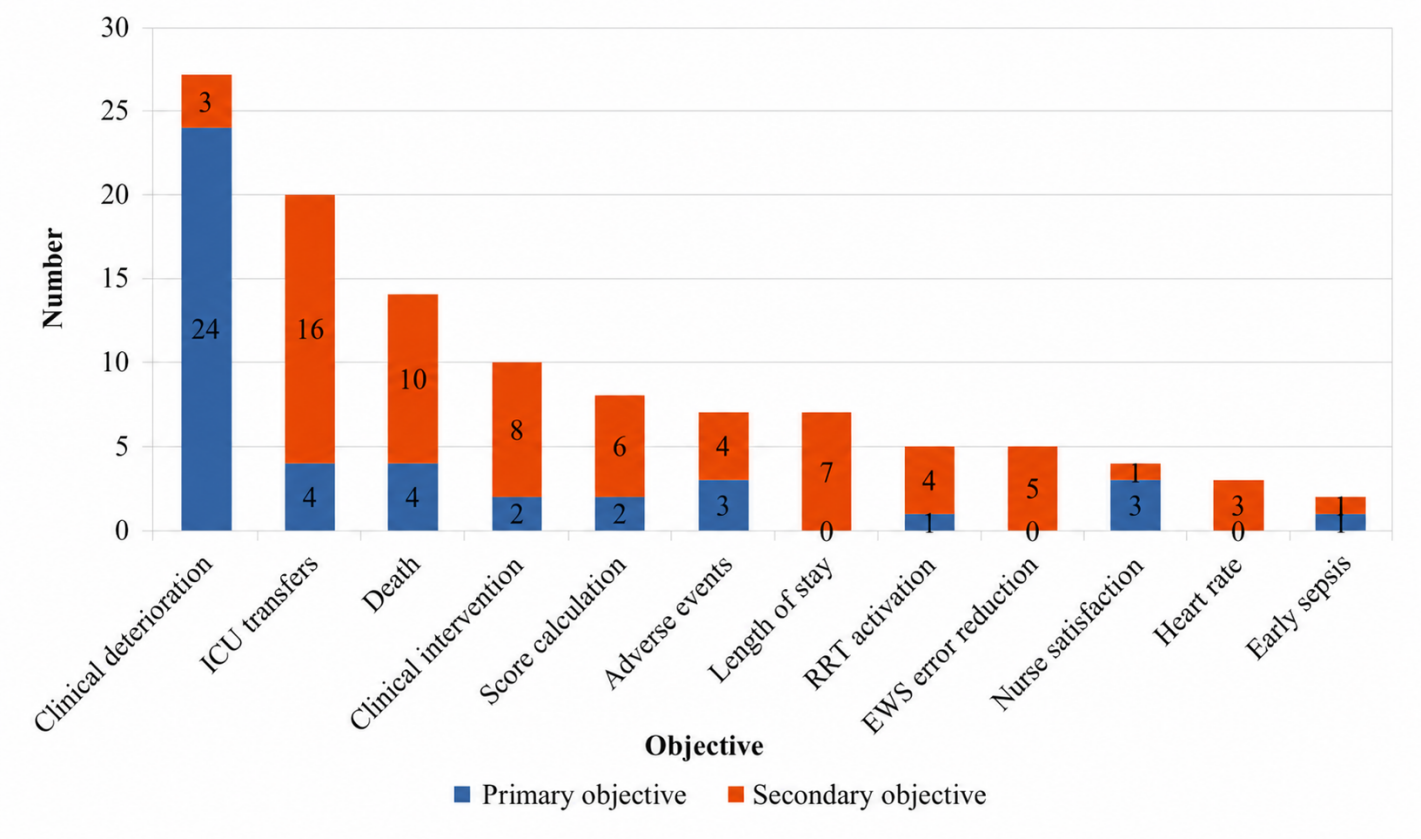
Frequency of early warning/track-and-trigger systems to primary and secondary objectives. The total frequency exceeds the number of included studies, as some studies reported multiple primary or secondary objectives. EW/TTS: early warning/track-and-trigger systems; ICU: intensive care unit; RRT: rapid response team.

The primary objectives of EW/TTS were CD detection in 24 of 44 reported primary objectives (54.5%), ICU transfer in 4 of 44 (9.1%), death in 4 of 44 (9.1%), and other objectives in 12 of 44 (27.3%). The secondary objectives were ICU transfer in 16 of 68 reported secondary objectives (23.5%), death in 10 of 68 (14.7%), clinical intervention in 8 of 68 (11.8%), length of stay in 7 of 68 (10.3%), and other objectives in 27 of 68 (39.7%; Figure 2).

To detect and predict CD, the EW/TTS used various clinical indexes, such as VS, assessment scores, clinical conditions, diagnostic results, demographics, and specialized measurements. VS parameters such as HR, RR, SpO_2_, BT, and BP, along with measurements such as stroke volume and cardiac index, dominate the landscape in 29 of 67 reported clinical indexes (43.3%), underscoring their critical role in patient monitoring. Assessment scores (EWS, pediatric EWS, patient status index, AVPU, and modified EWS) accounted for 13 of 67 (19.4%), highlighting their significance in evaluating patients’ conditions. Clinical conditions, such as consciousness, blood infections, and pain level, accounted for 9 of 67 (13.4%), underscoring the role of specific symptoms. Diagnostic results (6 of 67, 9%), demographics and history (6 of 67, 9%), and specialized measurements (4 of 67, 6%) were less frequent but still integral to comprehensive patient assessment. In summary, EW/TTS majorly relied on VS in CD detection or prediction (Figure S3 in Multimedia Appendix 2).

### Applied Technology, Algorithms, and Automated Features

According to ISHO’s adapted CMM [27], 18 of the 43 implemented EW/TTS (41.9%) were classified at the measured automation level. At this level, the EW/TTS data analysis processes are systematically implemented and actively managed. Ongoing efforts are in place to develop and refine the system, with established mechanisms to measure performance, track progress, and ensure coordination among involved teams. The managed automation level, identified in 11 of 43 (25.6%) [8,44,45,47,48,53,56,58,65,69] studies, represented an intermediate stage of automation maturity in which EW/TTS processes were implemented in practice but had not yet been systematically documented or institutionalized.

**Figure 3.**
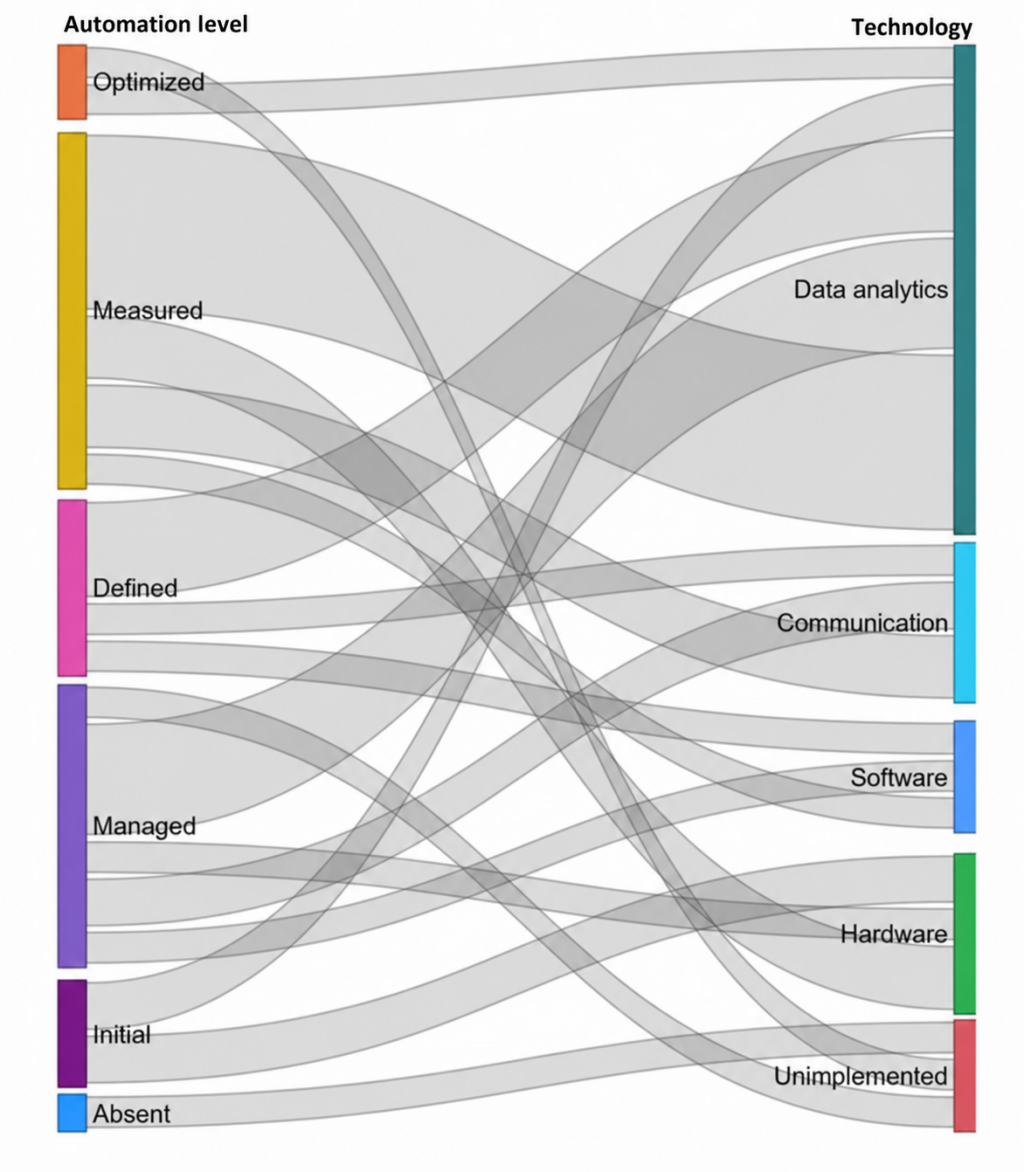
Automation levels (left) and technology domains of early warning/track-and-trigger systems (right; N=43). Data Collection did not appear on the right because n=0.

Seven of 43 (16.3%) studies [3,37,43,52,62-64] were classified at a defined automation level. Systematic, ongoing efforts to develop EW/TTS are underway; however, there is no comprehensive method to measure progress or ensure coordination. On the other hand, only 4 of 43 (9.3%) studies [32,40,59,68], 2 of 43 (4.7%) studies [35,36], and 1 of 43 (2.3%) studies [31] were classified as initial, optimized, and absent automation levels, respectively. The absent level represents a complete lack of automation capability, whereas the initial level is characterized by ad hoc and informal automation efforts. In contrast, the optimized level corresponds to the most advanced stage of automation, characterized by systematic, ongoing development of EW/TTS, supported by continuous quality improvement activities that align EW/TTS outcomes with the strategic objectives and performance metrics (Figure 3).

More than half of the included (24/43, 55.8%) studies [1,3,4,17,32,36,40,44-46,48-52,55,56,58,60,62-64,67,69] focused on data analytics technologies. Across the measured, defined, and managed automation levels, data analytics was the dominant technological focus in EW/TTS implementations. Data analytics included machine learning and AI, statistical analysis, visualization tools, and cloud services. Cloud services and visualization tools supported real-time dashboards for visual analytics. Predictive algorithms, including statistical and machine learning approaches, were identified in 11 of 43 (25.6%) studies [32,34,40-44,47,51,60,67].

Hardware technologies were identified in 7 of 43 (16.3%) studies [33,54,57,59,66,68,70], primarily comprising wearable devices and sensors. Wearable devices include HealthPatch [33], Hexiwear [38], ViSi Mobile [68], and waterproof patches [59], all of which are essential for continuously monitoring VS and other health metrics. These devices use sensors to record photoplethysmography (PPG) and other physiological data. Additionally, communication technologies accounting for 5 of 43 (11.6%) studies [34,41-43,47] use Bluetooth [32,36,52], Wi-Fi [33,52], and Bluetooth low energy (BLE) [32] to ensure seamless connectivity. Software technologies encompass mobile apps, eHandover, Node.js API, and eObs for remote health monitoring (Figure 3).

**Figure 4.**
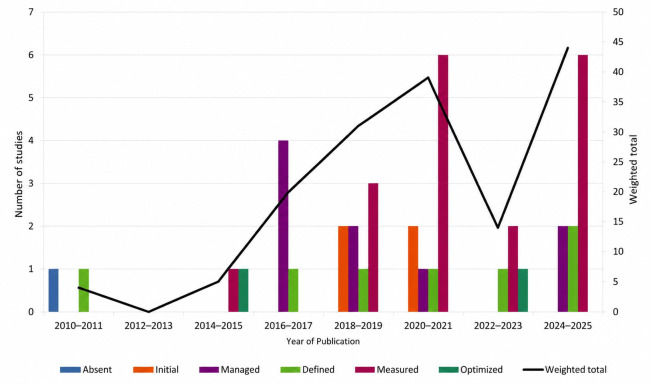
Trends in automation levels for the last 15 years.

The trend in EW/TTS automation indicated a clear long-term progression toward increased automation. During the early years (2010‐2015), there was minimal automation. Since 2016, there has been a consistent shift toward managed and measured automation levels. This transition drives a sharp rise in the weighted total, which peaks around 2020‐2021 and temporarily declines in 2022‐2023. We observed a strong rebound in 2024‐2025, with high levels of managed, defined, and measured automation signaling mature, data-driven, and continuously improving automation practices (Figure 4).

Data analysis using predictive algorithms, including statistical and machine learning approaches, occurred in only 11 of 43 (25.6%) [32,34,40-44,47,51,60,67] studies, all published in 2019 or later (Table 4). The applied algorithms included eCART [43], a multivariate probabilistic model [44], logistic regression-based models [34,41,42,47], logistic regression, decision trees, neural networks, and ensemble models [51], hybrid k-nearest neighbors least-squares support vector machines (SVMs) [40], natural language processing for text analysis, decision trees, and logistic regression [60], and a time-aware MARS model [67]. Furthermore, Chowdhury et al applied an SVM [32] for electrocardiogram (ECG) analysis and CD prediction within EW/TTS. Table 4 summarizes the predictive algorithms identified in the included studies, together with their reported data sources, prediction targets, time horizons, and performance metrics, where available. Thirty of 43 systems (69.8%) [3,17,33,34,36-38,41-44,47,49-51,53,54,56-58,60-65,67-70] had a data exchange feature and connectivity with other systems. Connectivity with EHRs was reported in 21 of 43 systems (48.8%) [3,17,34,37,41-44,47,50,51,53,56-58,61-64,67,69], wearable-device connectivity in 3 of 43 systems (7%) [33,38,54], and connectivity through a proprietary cloud platform in 1 of 43 systems (2.3%) [70]. Among connected systems, 5 of 43 (11.6%) [36,49,60,65,68] reported interoperability standards or communication platforms, including Fast Healthcare Interoperability Resource (FHIR), Health Level Seven (HL7)-based connectivity, and Ascom. Eight of 43 systems (18.6%) [1,4,31,32,35,40,48,59] had no connectivity, and connectivity was not specified in 5 of 43 systems (11.6%) [45,46,52,55,66].

The reported outcomes of EW/TTS were earlier warning in 14 of 70 reported outcomes (20%)**,** higher accuracy in 12 of 70 (17.1%)**,** lower specificity in 9 of 70 (12.9%)**,** more timely reaction in 7 of 70 (10), and reduced mortality in 6 of 70 (8.6%; Figure S4 in Multimedia Appendix 2).

**Table 4.** Predictive algorithms used in early warning/track-and-trigger systems reported data sources, targets, time horizons, and performance metrics.

Study or system	Algorithm type	Data sources	Prediction target	Time horizon	Performance reported
Youssef Ali Amer et al [40]	Hybrid KNN-LS-SVM^a^	Wearable vital-sign data	Clinical deterioration	1 h, 2 h, 3 h, dialysis: 1 h	MAPE^b^ 4.1%, 4.5%, and 5% for cardiology patients; real-time EWS feasible
Bassin et al [41]	Logistic regression	Demographics, vital signs, laboratory tests, temporal changes	ICU^c^ transfer, RRT^d^ activation, death	48 h	AUC 0.87 (prior validation); higher sensitivity than BTF^e^ up to 24 h; lower false alarms
Bell et al [42]	Logistic regression	Vital signs, laboratory values, EHR^f^	Clinical deterioration, death, ICU transfer, urgent surgery, or RRT activation	1‐48 h	AUC 0.87; sensitivity 0.474; specificity 0.972
Chowdhury et al [32]	SVM^g^ (polynomial kernel)	Wearable ECG^h^ from chest dry electrodes	STEMI/NSTEMI^i^ detection	10 s	Accuracy 97.4% (STEMI); 96.3% (NSTEMI)
Edelson et al [43]	eCARTv5	EHR^f^-based ward observations and clinical variables	Clinical deterioration, ICU transfer, death	24 h	AUROC^j^ 0.895; PPV^k^ 17.3% and 23.3%
Ehara et al [44]	Multivariate probabilistic AI model (VSI)	RR^l^, SpO₂^m^, BP^n^, HR^o^, temperature	ICU transfer, intubation, vasopressor use, composite outcome	24 h	AUC^p^ 0.767 (ICU transfer); 0.712 (intubation); 0.733 (vasopressor use); 0.647 (composite outcome)
Escobar et al [34]	Logistic-regression model; automated predictive model	EHR, laboratory tests, vital signs, neurologic status, severity of illness	Clinical deterioration, ICU transfer, death in hospital ward, 30-day mortality after alert	12 h	Sensitivity 49%; adjusted relative risk for 30-day mortality after alert 0.84 (95% CI 0.78‐0.90)
González et al [47]	Multivariable logistic regression	Age, sex/type of patient, HR, RR, temperature, supplemental O₂, GCS^q^	ICU transfer, death, composite outcome	8 h	AUC 0.832 (composite outcome); 0.832 (death); 0.723 (ICU transfer)
Huete-Garcia and Rodriguez-Lopez [51]	Logistic regression; decision trees; neural networks; ensemble models	Vital signs, laboratory test results	Critical events	NS^r^	AUROC 0.85 (logistic regression); 0.88 (decision trees); 0.92 (neural networks)
Rossetti et al [60]	NLP^s^, decision trees, and logistic regression	Nursing documentation, vital signs, administered medications, withheld medications, clinical notes	Clinical deterioration, RRT activation, cardiac arrest, sepsis, ICU transfer, death	42 h	Clinical deterioration detected 42 h earlier than MEWS
Verma et al [67]	Time-aware MARS model	Real-time EMR^t^ data, vital signs, prior scores, temporal changes	Clinical deterioration, death	NS	Sensitivity 53%; PPV 31%; PPV threshold 30%

a KNN-LS-SVM: k-nearest neighbors least-squares support vector machine.

b MAPE: mean absolute percentage error.

c ICU: intensive care unit.

d RRT: rapid response team.

e BTF: between the flags of clinical deterioration.

f EHR: electronic health record.

g SVM: support vector machine.

h ECG: electrocardiogram.

i ST-elevation myocardial infarction / Non-ST-elevation myocardial infarction detection.

j Area under the Receiver Operating Characteristic Curve.

k PPV: positive predictive value.

l RR: respiratory rate.

m Saturation of Peripheral Oxygen.

n BP: blood pressure.

o HR: heart rate.

p AUC: area under the curve.

q GCS: Glasgow Coma Scale.

r NS: not specified.

s NLP: natural language processing.

t EMR: electronic medical record.

### Quality Assessment of Studies

We critically appraised the quality of the studies using the adapted JBI checklist comprising eight criteria [39]. Most studies received a high proportion of “Yes” responses, suggesting good compliance, particularly in “System evaluation,” “Lessons learned,” “Characteristics,” “Post-implementation,” “Pre-implementation,” and “Implementation.” However, criteria related to “Development history” and “System failure” were less well reported. Only 19 of 43 (44.2%) studies [1,3,4,17,31,32,35,36,42,45,48,49,51,55-57,59,62,70] clearly addressed each of these items, whereas 24 of 43 (55.8%) studies [33,34,37,38,40,41,43,44,46,47,50,52-54,58,60,61,63-69] were rated as No, Unclear, or Not Applicable (Table 5).

**Table 5.** Quality assessment of early warning/track-and-trigger systems according to the Joanna Briggs Institute checklist (N=43).

Criteria	Quality index			
	Yes, n (%)	No, n (%)	Unclear (%)	Not applicable, n (%)
Characteristics	36 (83.7)	3 (7)	1 (2.3)	3 (7)
Development history	19 (44.2)	16 (37.2)	6 (14)	2 (4.7)
Preimplementation	35 (81.4)	2 (4.7)	2 (4.7)	4 (9.3)
Implementation	31 (72.1)	4 (9.3)	4 (9.3)	4 (9.3)
System failure	19 (44.2)	13 (30.2)	7 (16.3)	4 (9.3)
Postimplementation	36 (83.7)	1 (2.3)	1 (2.3)	5 (11.6)
System evaluation	39 (90.7)	1 (2.3)	0 (0)	3 (7)
Lessons learned	37 (86)	2 (4.7)	2 (4.7)	2 (4.7)

## Discussion

### Principal Findings

This systematic review explored the automated features, algorithms, and technologies of electronic EW/TTS. We identified 43 systems that revealed the transition from manual charts to electronic data. We found only 16 countries implementing electronic EW/TTS; the majority were reported in the United States (15/43, 34.9%), the United Kingdom (6/43, 14%), and the Netherlands (6/43, 14%). This distribution may partly reflect differences in digital health readiness, interoperability capacity, and policy support for the implementation of standardized early warning systems. In the United States, near-universal hospital EHR adoption and increasing interoperable health information exchange may have facilitated the integration of EW/TTS into routine care workflows [72]. In the United Kingdom, the national standardization of NEWS/NEWS2 across the National Health Services probably made them easier to use by ensuring that escalation criteria, documentation practices, and clinical training were consistent [73]. In the Netherlands, sustained national efforts to promote eHealth and electronic health data exchange, including the proposed Wegiz framework, may likewise have strengthened readiness for digitally enabled monitoring and alerting systems [74]. In combination, these factors may have made it easier for EW/TTS to be developed, implemented, and published than in places with lower digital maturity or less standardized escalation frameworks.

Nevertheless, we assume that many countries still consider electronic EWS calculations or have not yet reported their EW/TTS. Since 2016, electronic EW/TTS have advanced gradually through integration with machine learning and AI models, particularly in critical care and emergency medicine [19].

The automation levels of electronic EW/TTS ranged from electronic VS monitoring to real-time risk score prediction. Current EW/TTS largely focus on EWS calculation [2], with only a limited proportion of the included studies applying predictive algorithms to support earlier detection of deterioration. However, shorter prediction intervals and more accurate models are still needed. Although several tools to predict CD already use deep learning [75], they are not yet integrated into current EW/TTS.

The adapted CMM categories reflected differences in how automation was implemented and governed in practice. Managed systems generally showed operational automation in routine use but limited standardization or performance tracking, whereas measured systems were supported by explicit evaluation, outcome monitoring, or both. From a clinical perspective, this distinction is important because more mature automation may support better workflow integration, greater transparency of system performance, and more reliable decision support, even when the underlying predictive intelligence remains limited.

**Figure 5.**
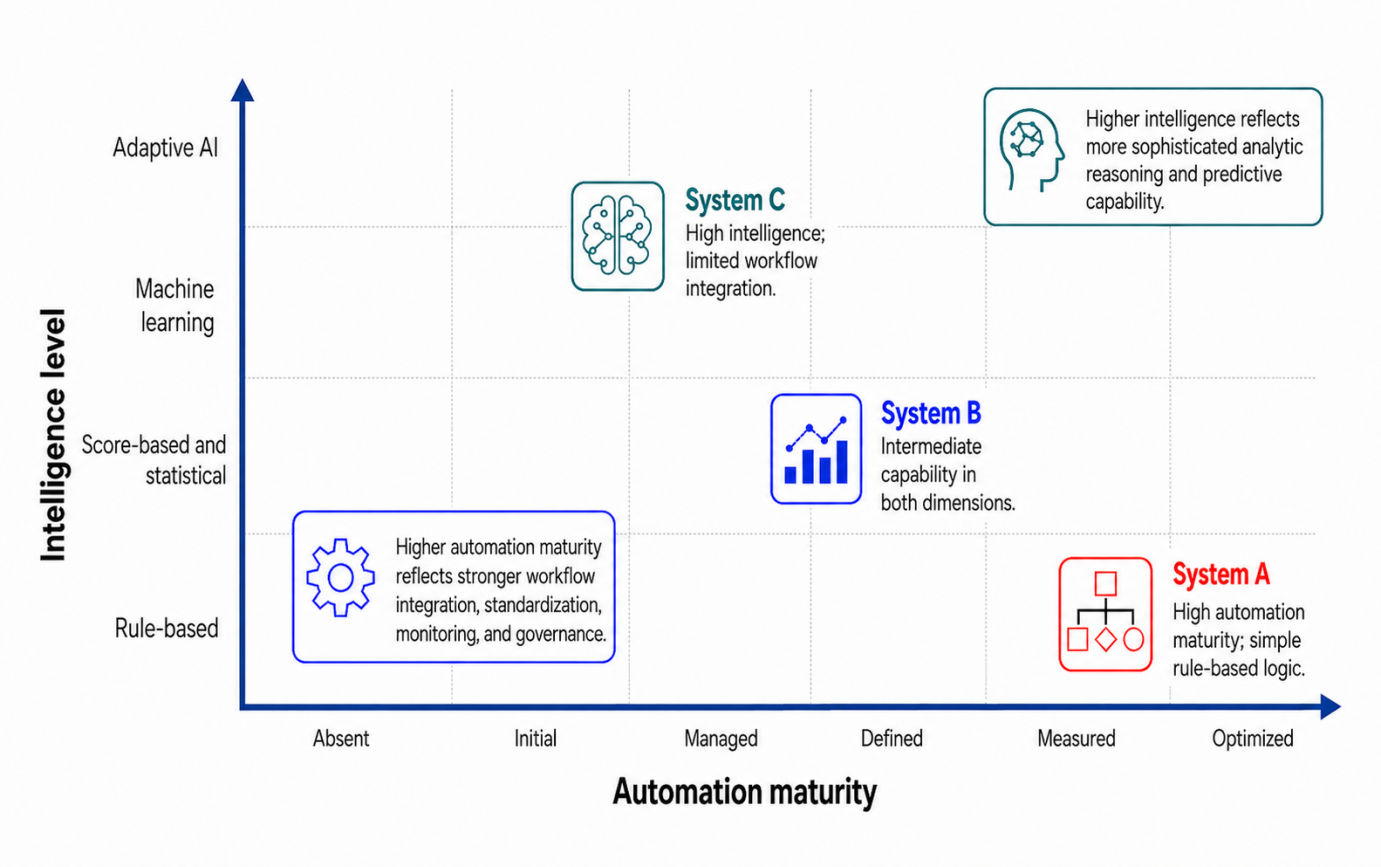
Conceptual two-dimensional framework of early warning/track-and-trigger systems automation maturity and intelligence. The horizontal axis shows automation maturity based on the adapted capability maturity model, and the vertical axis shows intelligence level from rule-based logic to adaptive AI. The framework highlights that automation maturity and algorithmic intelligence are related but distinct dimensions.

Automation maturity and algorithmic intelligence should not be interpreted as equivalent. In this review, the adapted CMM reflected the maturity of workflow automation, implementation, integration, and performance monitoring, whereas intelligence referred to the sophistication of the analytic approach, ranging from automated rule-based score calculation to statistical, machine learning, and more advanced AI-based models. Accordingly, a system could achieve a relatively advanced automation level through structured data acquisition, score calculation, alert delivery, and outcome monitoring without necessarily using machine learning. Conversely, the presence of predictive or AI-based models did not by itself indicate mature workflow integration (Figure 5).

The distinction between managed and measured automation also had practical implications beyond technical classification. Managed systems generally indicated that core automation functions were operational in routine care, but they often showed limited evidence of standardized evaluation, feedback, or outcome monitoring. By contrast, measured systems were supported by explicit assessment of performance, outcome tracking, or both. From a patient-safety perspective, this distinction is important because systems with measured automation may provide greater transparency regarding alert behavior, reliability, and unintended consequences. From a workflow perspective, measured systems may also be better positioned to support integration into escalation pathways and more consistent clinical use. In this sense, higher automation maturity may strengthen decision-making reliability not only through automation itself but through monitoring, validation, and governance of the automated process. Although the heterogeneity of study designs, outcomes, and performance metrics prevented a formal comparative analysis of maturity level against effectiveness, the reviewed evidence suggested that more mature systems were more often associated with structured evaluation, earlier warning, and workflow-oriented implementation features. At the same time, outcome trade-offs such as false alarms and lower specificity remained important, indicating that higher maturity should not be interpreted as uniformly better performance across all settings.

Predictive algorithms in the reviewed studies were primarily used to stratify patients according to their risk of CD, ICU transfer, AEs, or death [32,40,60]. For example, Chowdhury et al [32] reported an accuracy of 97.4% using SVM-based ECG analysis in a real-time wearable system. In this case, the high accuracy reflects the model’s ability to identify ECG patterns associated with myocardial infarction within a 10-second analysis window. This highlights that integrating AI-based analysis into EW/TTS enables real-time early warning of CD, extending beyond traditional EWS calculation. Preventive care and CD control aim not only at CD detection or prediction [76] but also at improving the quality of care, such as EWS error reduction [64], more timely reaction, and better decision-making for patient transfer to ICU, activating RRT, or optimizing the length of hospitalization [16]. Research in critical care has shifted the focus of EW/TTS’s outcomes from mortality reduction toward quality improvement.

Most EW/TTS operate in hospitals, especially in surgery wards (Figure S2 in Multimedia Appendix 2). Only 2 studies [32,38] considered CD detection in smart cars or in older adult care facilities. However, turning our medical systems from a curative to a preventive approach is essential [20]. Home care, particularly for older adults after hospital discharge or those living alone, increasingly relies on electronic EW/TTS to ensure safety monitoring within the private environment [77]. Deserno has already documented the general idea of transforming such places into a diagnostic space [20], and Wang et al [78] provided a comprehensive review on unobtrusive health monitoring in smart homes. Bringing this preventive approach to prehospital care may enhance the overall health care level and reduce AE, such as heart or respiratory failure, which are often precursors to death [79].

Gerry et al [16] assessed the efficacy of manual systems, whereas our systematic review evaluated electronic systems. The main reported outcomes of EW/TTS evaluation included early warning, high accuracy, and lower specificity (Figure S4 in Multimedia Appendix 2). These outcomes were generally positive, primarily for accurate, timely decision-making. Despite these positive results, electronic EW/TTS still need to improve through automation using AI technology.

Several studies reported the use of deep learning or machine learning to calculate predictive EWS [80,81]. The predictive models generated higher-level knowledge to support clinical decision-making. Hence, clinicians should replace the EWS calculation based on charts [2] with valid predictive models using AI methods [75]. Scores from charts indicate CD at the point of care, whereas the predicted value provides a forecast of CDs, which gives the nurse enough time to support the patient and activate RRT quickly. However, directly comparing algorithm performance across studies remains challenging because of substantial heterogeneity in clinical settings, input variables, prediction targets, and reported outcome measures. Cross-study comparability remained limited because the included EW/TTS differed substantially in clinical setting, prediction target, time horizon, and reported performance metrics. Prediction targets included CD, ICU transfer, AEs, and mortality; time horizons ranged from near-real-time alerts to forecasts several hours in advance; and performance was reported inconsistently using measures such as AUC, sensitivity, specificity, and PPV. As a result, the reported performance values should be interpreted within the context of each individual study rather than as directly comparable measures of superiority across systems or settings.

For instance, the system described by Alam et al [8] predicted EWS earlier based on different clinical, demographic, and physiological VS. The output alerted, reminded, and notified users based on their roles to ensure more sensitive, accurate, and timely actions.

Besides the remarkable advancements and achievements in AI, data analytics, cloud computing, and mobile technologies, significant improvements have occurred. Furthermore, high-speed internet, the Internet of Things, and standardized protocols facilitated seamless data exchange. Therefore, the full potential of AI and data analytics in electronic EW/TTS has not yet been realized [79].

### Comparison With Prior Work

Recent reviews have reported limited predictive performance of traditional aggregate-weighted EW/TTS [82]. Muralitharan et al [83] demonstrated that machine learning-based EW/TTS outperformed the conventional risk scoring approach in predicting CD. These findings align with the results identified in our review, which indicate a growing shift toward an automated EW/TTS approach.

**Figure 6.**
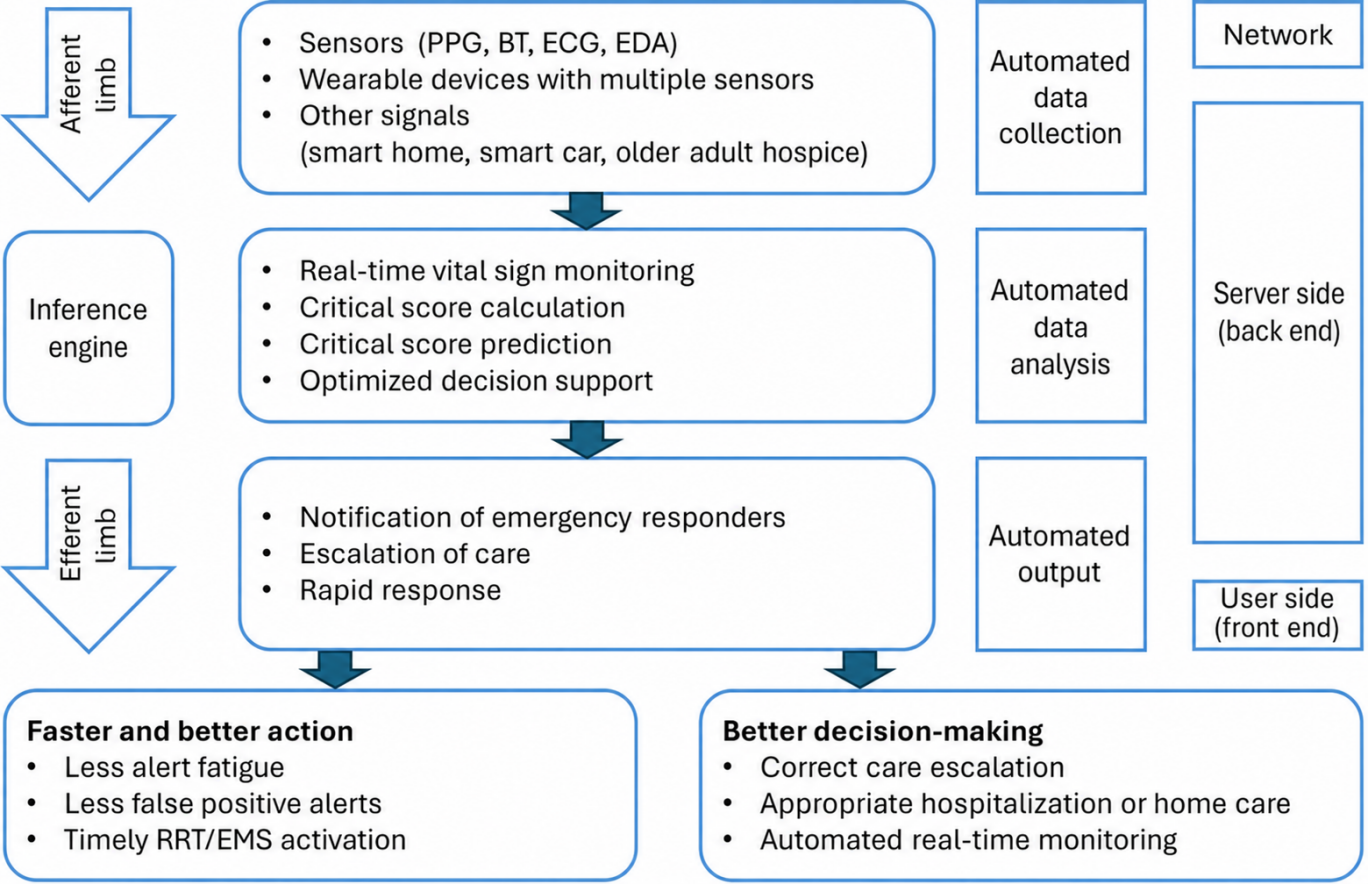
Intelligent early warning/track-and-trigger systems use modern technology for data collection, analysis, and output generation to improve decision-making and actions. BT: body temperature; ECG: electrocardiogram; EDA: electrodermal activity; EMS: emergency medical service; PPG: photoplethysmography; RRT: rapid response team.

Despite this advancement, the literature provides a limited synthesis of the extent of automation across EW/TTS components, particularly regarding data acquisition, algorithmic processing, and alert generation. Our review identified key enabling technologies and mapped their implementation across EW/TTS components. Based on the synthesized evidence, we propose a conceptual framework describing intelligent EW/TTS integration across three functional domains: automated data collection (afferent limb), inference and predictive analytics (inference engine), and automated output (efferent limb). In this conceptual model, the afferent limb is responsible for automated data acquisition, whereas the inference engine performs analytic functions such as VS interpretation, critical score calculation, and predictive modeling. The efferent limb is responsible for output functions, including alert generation, escalation, and communication to clinicians or RRTs (Figure 6). Intelligent EW/TTS integrates automated data acquisition within the afferent limb. This enables an embedded inference engine to continuously monitor VS, calculate and predict EWS, and apply advanced analytics to support actionable clinical decision-making. The inference engine links the afferent limb to the efferent limb by generating trigger outputs when predefined thresholds are exceeded. The efferent limb generates timely alerts to clinicians and RRTs, coordinates escalation pathways, and facilitates prompt intervention. By accelerating recognition and enhancing informed decision-making, these systems may improve patient outcomes. We therefore suggest that future intelligent EW/TTS should align both limbs with this model [11] and incorporate automation together with smart features. Sensors and wearable technologies for VS data collection may improve data quality. Research has shown that patch sensors provide higher precision for HR and RR [1].

The quality appraisal findings also affected the strength of evidence in this review. While most studies adequately described system characteristics, implementation, and evaluation, many provided limited information on development history and system failure. This gap makes it harder to assess how mature, reproducible, and thoroughly tested a system was before deployment. It also makes it more difficult to identify technical flaws, safety risks, and unintended effects that could arise during real-world use. As a result, the evidence may overemphasize reported benefits while underrepresenting implementation challenges and system-level risks. Readers should consider this limitation when interpreting the results.

### Limitations

However, this review also has limitations. First, most of the studies included in this review were conducted in hospital settings, which may limit the generalizability of the findings to nonclinical environments. Second, the collection of VS using wearable devices poses challenges, as some wearable monitoring systems still require further clinical evaluation and validation before broader adoption [52,59,68]. This may affect measurement accuracy and reliability. Third, regulatory and ethical factors, as well as the complexity of the health care system, may constrain widespread adoption of the EW/TTS. We also did not conduct a formal comparative analysis of country-level health system characteristics, including digital infrastructure readiness, EHR adoption, interoperability maturity, and national policy frameworks for early warning implementation, which may partially elucidate the concentration of reported EW/TTS in a limited number of countries.

Finally, the possible outcomes of implemented EW/TTS may include no change, statistically insignificant effects, or inconclusive results. However, we did not extract these outcomes because they were outside the scope of this review, which may limit the generalizability of our findings.

### Future Directions

Future research should focus on collecting high-quality, real-time VS data across diverse settings beyond hospitals, including smart home and in-vehicle environments, to improve the validity and accuracy of predictive models with acceptable sensitivity and specificity. The use of clinically validated wearable devices and the development of a standardized data collection framework may further improve model accuracy and reliability. Furthermore, adopting modern technologies such as smart devices, cloud computing, AI, robotics, and the Internet of Medical Things can revolutionize EW/TTS. AI technology powers intelligent EW/TTS to enhance decision-making and automate interventions. These advances offer opportunities to leverage modern technologies for transformative applications in health care, ultimately enhancing the quality of care and saving lives. As EW/TTS increasingly integrate AI-driven analytics and decision support, their deployment must also be evaluated within the context of evolving regulatory and digital health safety frameworks. Recent FDA guidance on AI-enabled device software functions and clinical decision support software emphasizes the need for clinical AI systems to be transparent, to undergo lifecycle oversight, to support performance monitoring, and to enable safe model updates.

### Conclusions

Current EW/TTS primarily focused on patient monitoring in hospital surgery wards and most commonly demonstrated measured automation. The primary objective of EW/TTS was to detect CD, while ICU transfers and mortality prediction were the most common secondary objectives. EW/TTS predominantly relied on VS and assessment scores to detect and predict CD. The included studies primarily used data analytics technologies. More than half of EW/TTS featured data exchange capabilities and connectivity with other systems. The primary reported outcomes of EW/TTS implementation included early warning, high accuracy, and lower specificity.

## Supplementary material

10.2196/58233 Multimedia Appendix 1 Search strategies.

10.2196/58233 Multimedia Appendix 2 Figures on study selection and EW/TTS analyses.

10.2196/58233 Multimedia Appendix 3 Critical appraisal checklist for electronic system.

10.2196/58233 Checklist 1 PRISMA checklist.

## Abbreviations

AE: adverse event
AVPU: alert, verbal, pain, and unresponsive
BLE: Bluetooth low energy
CD: clinical deterioration
CMM: capability maturity model
ECG: electrocardiogram
EHR: electronic health record
EW/TTS: early warning/track-and-trigger systems
EWS: Early Warning Score
FHIR: Fast Healthcare Interoperability Resources
HL7: Health Level Seven
HR: heart rate
ICU: intensive care unit
ISHO: Informatics-Savvy Health Organization
JBI: Joanna Briggs Institute
PPG: photoplethysmography
PRISMA: Preferred Reporting Items for Systematic Reviews and Meta-Analyses
RR: respiratory rate
RRT: rapid response team
SDC: Study Design Classification
SpO_2_: blood oxygen saturation
SVM: support vector machine
VS: vital sign
