# Supplementary material for: Automated Features, Algorithms, and Technologies of Electronic Early Warning/Track-and-Trigger Systems: Systematic Review
Source: J Med Internet Res. 2026 Aug 10;28:e58233. doi: 10.2196/58233 (PMC13456532; doi:10.2196/58233)
Supplement: Supplementary file 1 [file jmir-v28-e58233-s001.docx]

**Multimedia Appendix 1. Search strategies**

| **Databases** | **Search strategy** | **Identified records** |
| --- | --- | --- |
| **PubMed** | ("patient deterioration"[tiab] OR  "clinical deterioration"[tiab])  **AND**  (instability[tiab] OR  "failure to rescue"[tiab] OR  "early warning"[tiab] OR  "track and trigger"[tiab] OR  decompensation[tiab] OR  "heart attack"[tiab] OR "heart failure"[tiab] OR  "pulmonary failure"[tiab] OR  "renal failure"[tiab] OR  sepsis[tiab] OR  "care escalation"[tiab] OR  triage[tiab] OR  emergency[tiab] OR  warn*[tiab])  **AND**  (system*[tiab] OR  automat*[tiab] OR  smart*[tiab] OR  wearable*[tiab] OR  "internet of thing*"[tiab] OR  digital*[tiab] OR  signal*[tiab] OR  sensor*[tiab] OR  intelligen*[tiab] OR  prediction[tiab] OR  "expert system"[tiab]) | 403 |
| **Scopus** | (  TITLE-ABS-KEY("patient deterioration")  OR TITLE-ABS-KEY("clinical deterioration")  )  AND  (  TITLE-ABS-KEY(instability)  OR TITLE-ABS-KEY("failure to rescue")  OR TITLE-ABS-KEY("early warning")  OR TITLE-ABS-KEY("track and trigger")  OR TITLE-ABS-KEY(decompensation)  OR TITLE-ABS-KEY("heart attack")  OR TITLE-ABS-KEY("heart failure")  OR TITLE-ABS-KEY("pulmonary failure")  OR TITLE-ABS-KEY("renal failure")  OR TITLE-ABS-KEY(sepsis)  OR TITLE-ABS-KEY("care escalation")  OR TITLE-ABS-KEY(triage)  OR TITLE-ABS-KEY(emergency)  OR TITLE-ABS-KEY(warn*)  )  AND  (  TITLE-ABS-KEY(system*)  OR TITLE-ABS-KEY(automat*)  OR TITLE-ABS-KEY(smart*)  OR TITLE-ABS-KEY(wearable*)  OR TITLE-ABS-KEY("internet of thing*")  OR TITLE-ABS-KEY(digital*)  OR TITLE-ABS-KEY(signal*)  OR TITLE-ABS-KEY(sensor*)  OR TITLE-ABS-KEY(intelligen*)  OR TITLE-ABS-KEY(prediction)  OR TITLE-ABS-KEY("expert system")  ) | 606 |
| **Web of Sciences** | TS=("patient deterioration" OR  "clinical deterioration")  **AND**  TS=( instability OR  "failure to rescue" OR  "early warning" OR  "track and trigger" OR  decompensation OR  "heart attack" OR  "heart failure" OR  "pulmonary failure" OR  "renal failure" OR  sepsis OR  "care escalation" OR  triage OR  emergency OR  warn*)  **AND**  TS=(system* OR  automat* OR  smart* OR  wearable* OR  "internet of thing*" OR  digital* OR  signal* OR sensor* OR  intelligen* OR  prediction OR  "expert system") | 172 |
