# Supplementary material for: Automated Features, Algorithms, and Technologies of Electronic Early Warning/Track-and-Trigger Systems: Systematic Review
Source: J Med Internet Res. 2026 Aug 10;28:e58233. doi: 10.2196/58233 (PMC13456532; doi:10.2196/58233)
Supplement: Supplementary file 2 [file jmir-v28-e58233-s002.docx]

**Multimedia Appendix 2. Figures on study selection and EW/TTS analyses.**

Figure S1 in Multimedia Appendix 4. Flowchart of study identification, screening, eligibility assessment, and included records.

Figure S2 in Multimedia Appendix 4. Frequency of EW/TTS according to study settings. The total frequency exceeds the number of included studies, as some studies reported multiple settings.

Figure S3 in Multimedia Appendix 4. Frequency of clinical indexes for CD detection and prediction. The total frequency exceeds the number of included studies, as some studies used multiple clinical index.

Figure S4 in Multimedia Appendix 4. The reported outcomes of applying EW/TTS. The total frequency exceeds the number of included studies, as some studies reported multiple outcomes.
