# Supplementary material for: Automated Features, Algorithms, and Technologies of Electronic Early Warning/Track-and-Trigger Systems: Systematic Review
Source: J Med Internet Res. 2026 Aug 10;28:e58233. doi: 10.2196/58233 (PMC13456532; doi:10.2196/58233)
Supplement: Supplementary file 3 [file jmir-v28-e58233-s003.docx]

**Multimedia Appendix 3. Critical Appraisal Checklist for Electronic System**

Reviewer: _____________________
Date: _____________________

Author: ______________________
Year: ________
Record Number: ________

| **Criteria** | **Category** | **Yes** | **No** | **Unclear** | **Not Applicable** |
| --- | --- | --- | --- | --- | --- |
| 1. Were the system’s characteristics (e.g., architecture, features) clearly described? | System characteristic | ⬜ | ⬜ | ⬜ | ⬜ |
| 2. Was the system’s development history (e.g., design, testing phases) clearly described and presented as a timeline? | Development history | ⬜ | ⬜ | ⬜ | ⬜ |
| 3. Was the system's status or condition at the time of implementation clearly described? | Pre-implementation | ⬜ | ⬜ | ⬜ | ⬜ |
| 4. Was the system's implementation process (e.g., setup, training, integration) clearly described? | Implementation process | ⬜ | ⬜ | ⬜ | ⬜ |
| 5. Were system errors, failures, or unanticipated events identified and described? | System failure | ⬜ | ⬜ | ⬜ | ⬜ |
| 6. Was the system's performance post-implementation clearly described? | Post-implementation | ⬜ | ⬜ | ⬜ | ⬜ |
| 7. Were the assessment methods and results used to evaluate the system clearly described? | System evaluation | ⬜ | ⬜ | ⬜ | ⬜ |
| 8. Does the report provide lessons learned regarding the system’s design, implementation, or performance? | Lessons  learned | ⬜ | ⬜ | ⬜ | ⬜ |

**Overall Appraisal**:

- Include ⬜
- Exclude ⬜
- Seek further information ⬜

**Comments (including reason for exclusion)**:

### **TOOL GUIDANCE**

**Answers:** Yes, No, Unclear, or Not Applicable

1. **Were the system’s characteristics (e.g., architecture, functionalities) clearly described? 1**
   - Does the report clearly describe the system’s key characteristics, such as platform (e.g., web-based, mobile), functionalities, and technical details? The context or setting in which the system is used may also be included (e.g., hospital, emergency department, outpatient clinic).
2. **Was the system’s development history clearly described and presented as a timeline?2**
   - A good system report will clearly describe the history of the system’s development, including the design process, testing phases, and any relevant iterations or updates. This should include past interventions and their outcomes (e.g., improvements made based on testing feedback).
3. **Was the system’s condition at the time of implementation clearly described?**
   - The report should describe the state of the system at the time of its implementation, including the stability of the system, any known limitations, and readiness for deployment. It should also mention any unique features or potential issues that were identified.
4. **Was the system’s implementation process clearly described?5**
   - It is important to clearly describe how the system was implemented, including any installation processes, integration with existing systems, training provided to users, and any challenges encountered. This helps ensure that other institutions can understand how to implement the system in their own settings.
5. **Where system errors, failures, or unanticipated events identified and described?**
   - As with any complex system, failures or errors can occur. It is important that these events are documented, particularly in cases where the system is new or innovative. Any unanticipated issues that arise should be clearly described, as they may offer valuable insights for future system development.
6. **Was the system’s performance post-implementation clearly described?**
   - A good system report should describe the system’s performance after it was implemented, including the presence or absence of any issues, and whether the system met its performance goals (e.g., improving workflow, reducing errors). Visual aids like graphs or charts can help in conveying these outcomes clearly.
7. **Were the evaluation methods and results used to assess the system clearly described?**
   - The report should provide sufficient information to understand how the system was evaluated. This includes descriptions of the tests, simulations, or performance metrics used (e.g., response time, error rate). Visual aids like screenshots or diagrams may also be presented to convey the information clearly.
8. **Does the system report provide lessons learned?**
   - System reports should summarize key lessons learned from the development, implementation, and use of the system. These lessons can inform future system designs, deployment strategies, and user training. Practical guidance on how to avoid challenges or improve future iterations should also be included.

### **How to use this tool?**

This appraisal tool is designed to critically assess the quality of reports on the development, implementation, and performance of electronic systems. It helps reviewers evaluate whether the system is well-described, how it was implemented, and if it delivered on its intended goals, while also offering practical lessons for future improvements.
